# First-in-human exploratory trial assessing safety, feasibility, and efficacy of artificial protein (silk-elastin) in promoting healing in patients with meniscus injuries

**DOI:** 10.1038/s41598-025-88616-x

**Published:** 2025-02-07

**Authors:** Masakazu Ishikawa, Shunya Tsuji, Goki Kamei, Kyohei Nakata, Akinori Nekomoto, Naofumi Hashiguchi, Tomoyuki Nakasa, Atsuo Nakamae, Naosuke Kamei, Keiichiro Inoue, Shingo Kawabata, Keiko Ueda, Nobuo Adachi

**Affiliations:** 1 Department of Orthopedic Surgery, Faculty of Medicine, Kagawa University, Kagawa, Japan; 2 Department of Orthopedic Surgery, Graduate School of Biomedical and Health Sciences, Hiroshima University, Hiroshima, Japan; 3 Sanyo Chemical Industries, Kyoto, Japan; 4 Trarnslation Research Center for Medical Innovation, Kobe, Hyogo Japan; 5 Department of Orthopedic Surgery, Faculty of Medicine, Kagawa University, 1750-1 Ikenobe, Miki-cho, Kita-gun, Kagawa, 761-0793 Japan

**Keywords:** Meniscus tear, Arthroscopic meniscus repair, Artificial protein, Silk-elastin, Needle arthroscope, Knee osteoarthritis, Cartilage, Drug development

## Abstract

Meniscal tears, especially those in avascular regions, pose a significant risk for osteoarthritis if repair fails. While meniscal repair is the preferred method for preserving knee function, it often has a high failure rate in avascular zones. This study aimed to evaluate the safety and potential efficacy of silk-elastin (SE), an artificial protein with wound-healing properties, for enhancing meniscal repair. Eight patients with meniscal tears in avascular areas underwent arthroscopic repair followed by SE application, including cases of lateral and medial tears, discoid lateral meniscus, and bucket-handle tears. No adverse events or reactions were attributed to SE. At 3 months post-surgery, clinical outcomes and repair sites were evaluated using MRI and arthroscopy. Significant improvements were observed in Lysholm and visual analog scale scores (*P* < 0.05), with the knee injury and osteoarthritis outcome scores showing significant improvement in the symptom subscale. MRI findings indicated one patient with grade 1 healing, three with grade 2, and four with grade 3 (unhealed). Arthroscopically, six patients demonstrated completely healed menisci, while two showed incomplete healing; none were classified as “unhealed.” These findings suggest that SE is safe and may support meniscal healing in avascular zones, indicating its potential to improve repair outcomes.

## Introduction

Meniscal tears are among the most common injuries in orthopedics^1^. The menisci primarily function to provide joint stability and absorb shock^2,3^. Partial or total meniscectomy has shown that reduced meniscal tissue increases contact stress, accelerating degenerative changes in the knee^4–6^. Therefore, preserving the function of an injured meniscus through repair is crucial for preventing the onset or progression of knee osteoarthritis (KOA)^7^. Meniscal injury is a known trigger for secondary KOA. Given the increased longevity and high activity levels in the aging population, treating meniscal injuries with a focus on preventing secondary KOA has gained attention as a strategy to preserve knee joint function and slow osteoarthritis progression at a relatively young age.

Interest in developing augmentation techniques to enhance the healing and outcomes of meniscal repair surgeries is growing^8,9^. Advances in suturing devices have made meniscus suturing a feasible option; however, certain tear types, such as radial or bucket-handle tears, pose challenges for suturing and have limited healing potential^10,11^. In these cases, partial or complete meniscectomy may be performed instead of repair. Additionally, the high failure rate of meniscal repairs has raised concerns among patients and orthopedic surgeons, prompting discussions on innovative methods to improve meniscal healing and clinical outcomes^12,13^. One approach under consideration is the development of augmentation techniques to facilitate post-repair healing. Platelet-rich plasma and mesenchymal progenitor or stem cell therapies have been investigated as potential augmentation options^14–17^; however, issues related to biomedical regulations, equipment, and cost remain unresolved.

Silk-elastins (SEs), artificial proteins developed through genetic engineering and biological production methods, have been studied for their ability to promote tissue healing^18–20^. Previous basic and clinical studies have shown that SEs, particularly 47 K (P47K-WAS-MR) – a polymer containing 12 repeats of a sequence with four elastin-like motifs, a V-to-K-replaced elastin-like motif, three additional elastin-like motifs, and four silk-fibroin-like motifs – hold promising therapeutic potential for accelerating wound healing in animal models and clinical trials^21–23^. Additionally, artificial proteins, due to their off-the-shelf nature, do not require blood sampling or cell processing, posing fewer regulatory concerns for operating room use and aligning with regenerative medicine principles. Based on previous evidence, the therapeutic potential of SE, P47K-WAS-MR, for meniscus injury has been confirmed in a rabbit meniscus injury model^24^.

This study aimed to investigate the safety, technical feasibility, and exploratory efficacy of arthroscopic SE administration in achieving successful healing at the repair site in patients with meniscal tears. This single-arm, single-center phase I/II trial was designed to assess the potential of SE as an adjunct therapy for meniscal repair.

## Results

### Participants

Nine participants consented to participate in the trial; however, one patient was excluded because the P47K-WAS-MR gel was not applied, as the meniscal tear involved a red-red zone, a vascular-rich area observed arthroscopically during surgery. Ultimately, eight patients completed the treatment. Table 1 presents the participants’ demographics and clinical characteristics.

**Table 1:** Demographic and clinical characteristics of the patients.

Patient #	Age	Gender	Side	BMI	Diagnosis	Time to surgery (months)	Lesion location	Type of tear	Surgical procedure	Operation time (min)
1	20	F	R	23.4	LM tear	10	Mid to posterior	Complex, horizontal oblique	AIS: FastFix 2 sutures Scorpion: 2 sutures	78
2	27	M	R	26.9	LM tear	4	Mid	Horizontal & radial	IOS 3 sutures	76
3	38	M	R	25.5	LM tear	5	Anterior	Longitudinal-vertical	IOS 3 sutures	65
4	17	M	L	22.7	LM tear, DLM	13	Mid to posterior	Mid flap tear and posterior horizontal	Partial meniscectomy AIS: Scorpion: 2 sutures	79
5	17	M	R	17.5	MM tear	3	Mid to posterior	Longitudinal-vertical, BHT	IOS 11 sutures	107
6	47	F	R	18.3	MM tear	11	Posterior	Horizontal	AIS: Scorpion: 2 sutures	4
7	52	M	R	29.3	MM tear	14	Posterior	Horizontal	AIS FastFix 2 sutures	55
8	21	M	R	26.6	MM tear	5	Anterior to posterior	Longitudinal-vertical, BHT	AIS: FastFix 2 sutures IOS: 10 sutures	117

R: right; L: left; LM: lateral meniscus; DLM: discoid lateral meniscus; MM: medial meniscus; AIS: all-inside suture; IOS: inside-out suture; BHT: bucket-handle tear.

## Primary outcomes

### Feasibility

**Fig. 1:**
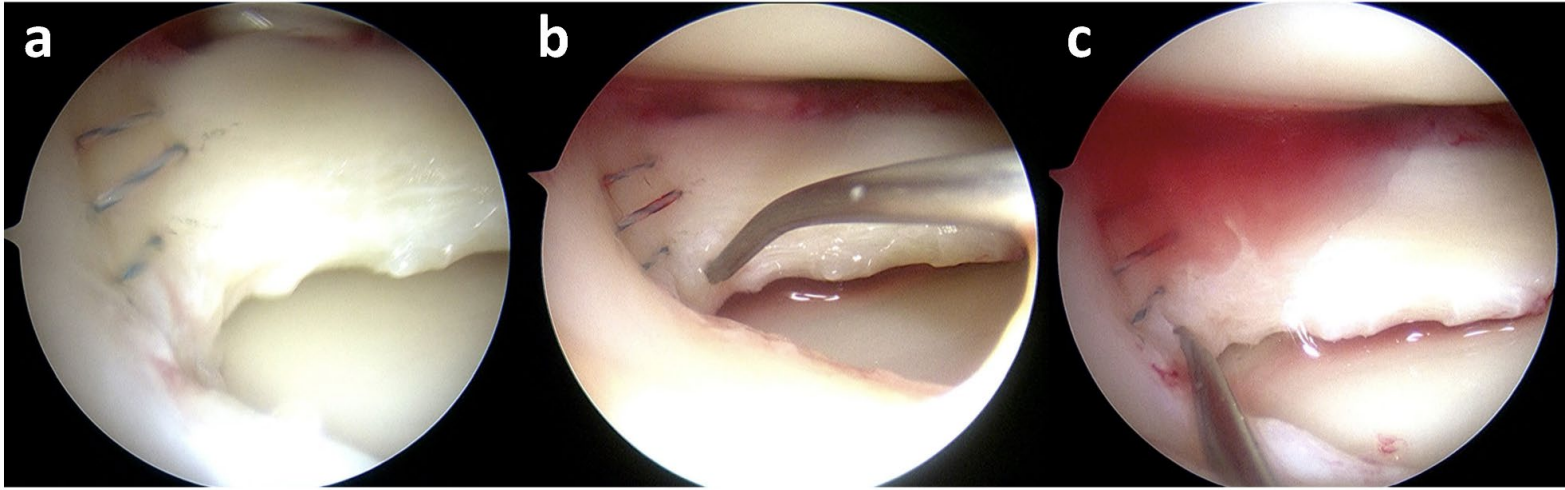
Meniscal repair and P47K-WAS-MR gel application. (**a**) Arthroscopic repair of a radial tear in the lateral meniscus. (**b**) A needle positioned at the repair site through the arthroscopic portal. (**c**) Application of the P47K-WAS-MR gel to the repair site.

The medial and lateral menisci were successfully repaired arthroscopically in all patients (Table 1). During surgery, the P47K-WAS-MR sponge was prepared in gel form and applied arthroscopically with a needle without complications (Fig. 1).

### Safety

No serious adverse events were observed during the study, and no device faults that could cause adverse events for patients or investigators were detected. Forty-two non-serious adverse events occurred among the eight patients (Table 2). The adverse events observed in three or more patients, in descending order of incidence, were joint pain (*n* = 7; 87.5%), increased C-reactive protein (CRP) levels (*n* = 5; 62.5%), treatment-related pain (*n* = 4; 50.0%), fever, and joint effusion (*n* = 3; 37.5% each). Arthralgia persisted for up to 2 days postoperatively, while CRP levels rose postoperatively but normalized by 8 weeks. Treatment-related pain subsided within 2 weeks, fever resolved within 2 days, and joint effusion improved within 2–6 weeks.

**Table 2:** Adverse events during follow up.

Adverse event	Number of cases (%)	Level of severity	Degree of seriousness	Outcome	Relation to the application
Treatment-related pain	4 (50.0)	Moderate	Non-serious	Recovered	Not related
Fever	3 (37.5)	Moderate	Non-serious	Recovered	Might be related
Back pain	2 (25.0)	Moderate	Non-serious	Recovered	Not related
Nausea	2 (25.0)	Moderate	Non-serious	Recovered	Might be related
Insomnia	1 (12.5)	Moderate	Non-serious	Recovered	Not related
Itchiness	1 (12.5)	Moderate	Non-serious	Recovered	Not related
Joint pain	7 (87.5)	Mild	Non-serious	Recovered	Not related
CRP elevation	5 (62.5)	Mild	Non-serious	Recovered	Not related
Joint effusion	3 (37.5)	Mild	Non-serious	Recovered	Not related
Postoperative anemia	2 (25.0)	Mild	Non-serious	Recovered	Might be related
Amylase elevation	2 (25.0)	Mild	Non-serious	Recovered	Not related
CPK elevation	2 (25.0)	Mild	Non-serious	Recovered	Not related
ALP elevation	2 (25.0)	Mild	Non-serious	Recovered	Might be related
Swelling	1 (12.5)	Mild	Non-serious	Recovered	Not related
ALT elevation	1 (12.5)	Mild	Non-serious	Recovered	Not related
AST elevation	1 (12.5)	Mild	Non-serious	Recovered	Not related
Eosinophil elevation	1 (12.5)	Mild	Non-serious	Recovered	Not related
Hypersensitive	1 (12.5)	Mild	Non-serious	Recovered	Not related
Hypertension	1 (12.5)	Mild	Non-serious	Recovered	Not related

CRP: C-reactive protein; ALP: alkaline phosphatase; ALT: alanine transaminase; AST: aspartate transferase.

Seventeen moderate adverse events were reported in six of eight patients (75.0%). The moderate adverse events occurring in three or more patients were treatment-induced pain (*n* = 4; 50.0%) and fever (*n* = 3; 37.5%). Additionally, all eight patients (100.0%) experienced 32 mild adverse events. The most common mild adverse events, in descending order of incidence, were treatment-induced joint pain (*n* = 7; 87.5%), increased CRP (*n* = 5; 62.5%), and joint effusion (*n* = 3; 37.5%).

## Secondary outcomes

### Efficacy

**Fig. 2:**
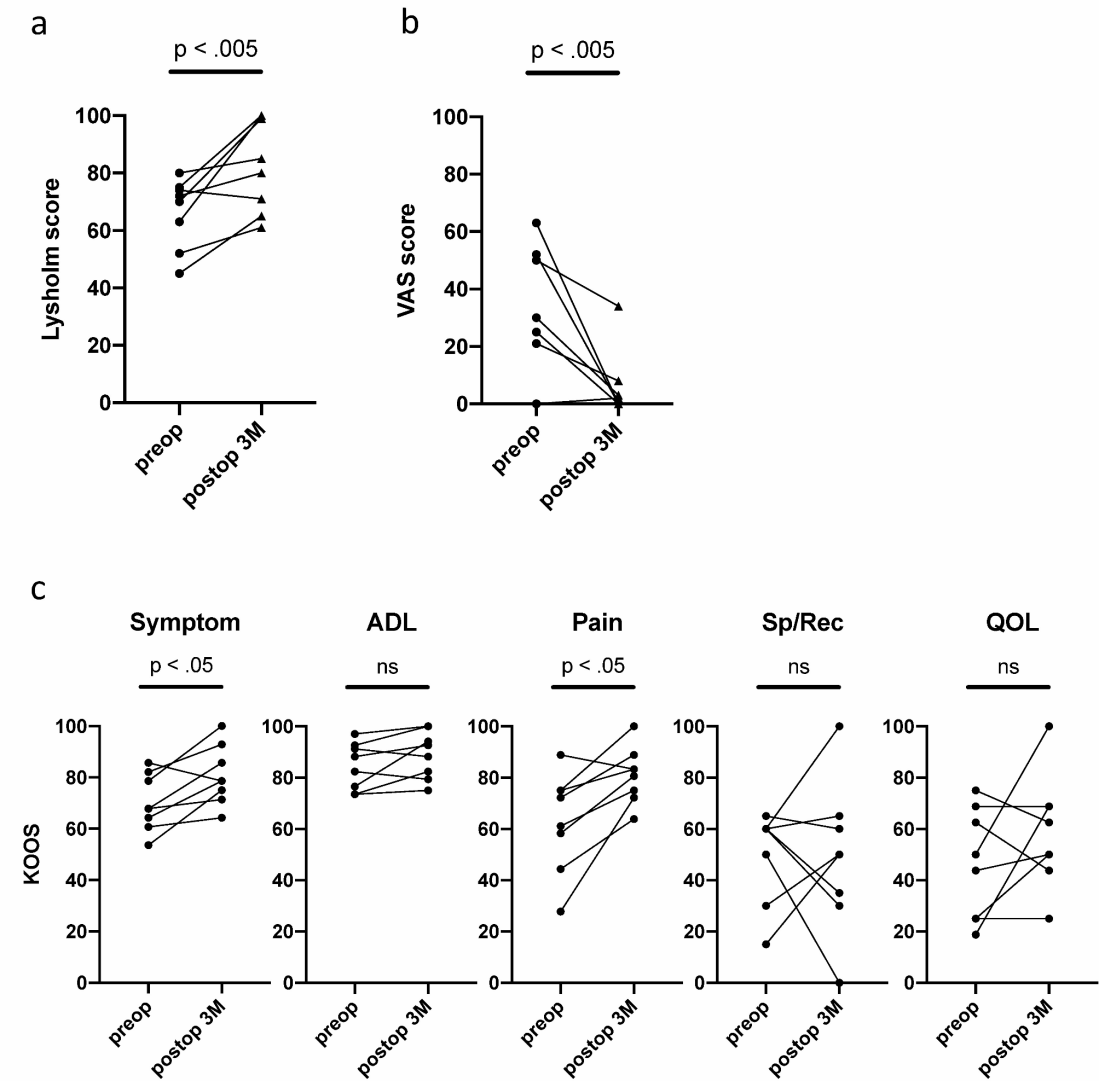
Secondary outcomes at 3 months post P47K-WAS-MR application. (**a**) Lysholm score, (**b**) VAS pain score, and (**c**) KOOS subscales.

The mean Lysholm score improved significantly from baseline after 3 months (preoperative score: 66.4 ± 12.2, postoperative score: 82.6 ± 16.0, *P* < 0.05) (Fig. 2a). The mean visual analog scale (VAS) score also showed significant improvement after 3 months (preoperative score: 38.0 ± 22.5, postoperative score: 5.9 ± 11.7, *P* < 0.05) (Fig. 2b). For the knee injury and osteoarthritis outcome score (KOOS), significant improvement was observed only in the symptom and pain subscales (Fig. 2c) (Table 3).

**Table 3:** Secondary outcome after 3 months of surgery – clinical scores.

Patient #	Lysholm score		VAS score		KOOS (S-A-*P*-Sp-Q)	
	Preop	Postop	Preop	Postop	Preop	Postop
1	70	99	25	0	67.8–97.0–72.2–60.0–75.0	85.7–100 – 88.9–30.0–62.5
2	52	61	63	0	82.1–82.4–61.1– 50.0–62.5	92.9–79.4–75.0–0.0–43.8
3	72	80	21	8	67.9–88.2–75.0–60.0–25.0	71.4–92.6–83.3–35.0–25.0
4	75	100	63	0	78.6–92.6–75.0–60.0–50.0	100–100 – 100–100 – 100
5	80	85	0	2	85.7–91.2–88.9–65.0–68.8	78.6–88.2–83.3–60.0–68.8
6	45	65	50	34	60.7–73.5–44.4–60.0–43.8	64.3–75.0–63.9–65.0–50.0
7	63	100	52	0	53.6–73.5–27.8–30.0–18.8	75.0–82.4–72.2–50.0–68.8
8	74	71	30	3	64.3–76.5–58.3–15.0–25.0	78.6–94.1–80.6–50.0–50.0

VAS: visual analog scale; KOOS: knee injury and osteoarthritis outcome score; S: symptom; A: activity of daily life; P: pain; Sp: sports and recreation; Q: quality of life; Preop: preoperative; Postop: postoperative.

### Evaluation of healing status using magnetic resonance imaging (MRI) at 3 months post-surgery

**Fig. 3:**
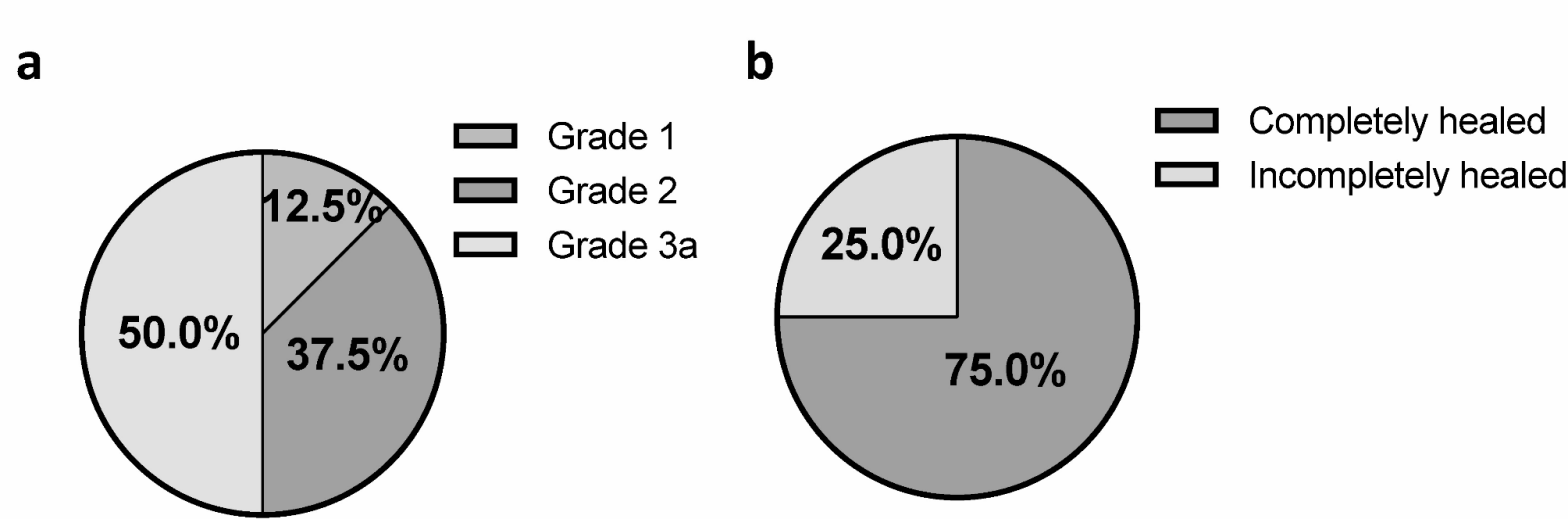
Secondary outcomes, including image assessments at 3 months post P47K-WAS-MR gel application. (**a**) MRI grading of healing and (**b**) arthroscopic evaluation of healing status from second-look arthroscopy.

None of the participants achieved grade 0 status. One patient was classified as grade 1, three as grade 2, and four (all grade 3a) as grade 3. This evaluation yielded a failure rate of 50.0% (95% confidence interval: 15.7, 84.3) (Fig. 3a). By contrast, in the surgeon’s own evaluation, five cases were classified as grade 2 (62.5%) and three as grade 3a (37.5%) (Table 4).

**Table 4:** Secondary outcome after 3 months of surgery – MRI and arthroscopic assessment.

Patient #	MRI grading		Second-look arthroscopic status		
	Surgeon’s evaluation	Central evaluation	Needle arthroscopy	Surgeon’s evaluation	Central evaluation
1	Grade3a	Grade2	-	Incompletely healed	Completely healed
2	Grade2	Grade1	-	Completely healed	Completely healed
3	Grade2	Grade2	Nanoscope®	Completely healed	Completely healed
4	Grade2	Grade3a	Needle Arthroscope®	Incompletely healed	Incompletely healed
5	Grade3a	Grade3a	Nanoscope®	Incompletely healed	Incompletely healed
6	Grade3a	Grade3a	Nanoscope®	Completely healed	Completely healed
7	Grade2	Grade2	Nanoscope®	Incompletely healed	Completely healed
8	Grade2	Grade3a	Nanoscope®	Completely healed	Completely healed

MRI: magnetic resonance imaging.

### Arthroscopic assessment of healing status at 3 months post-SE application

Needle arthroscopy was performed in six patients, while conventional arthroscopy was performed on eight. Based on conventional arthroscopy, six of eight participants were assessed as having complete healing, two as having incomplete healing, and none as unhealed, resulting in a failure rate of 0.0% (95% confidence interval: 0.0, 36.9) 3 months post-surgery with SE application (Fig. 3b). According to the surgeon’s evaluation, four cases were completely healed, and four were incompletely healed, with a failure rate of 0.0% (Table 4).

### Case presentations

**Fig. 4:**
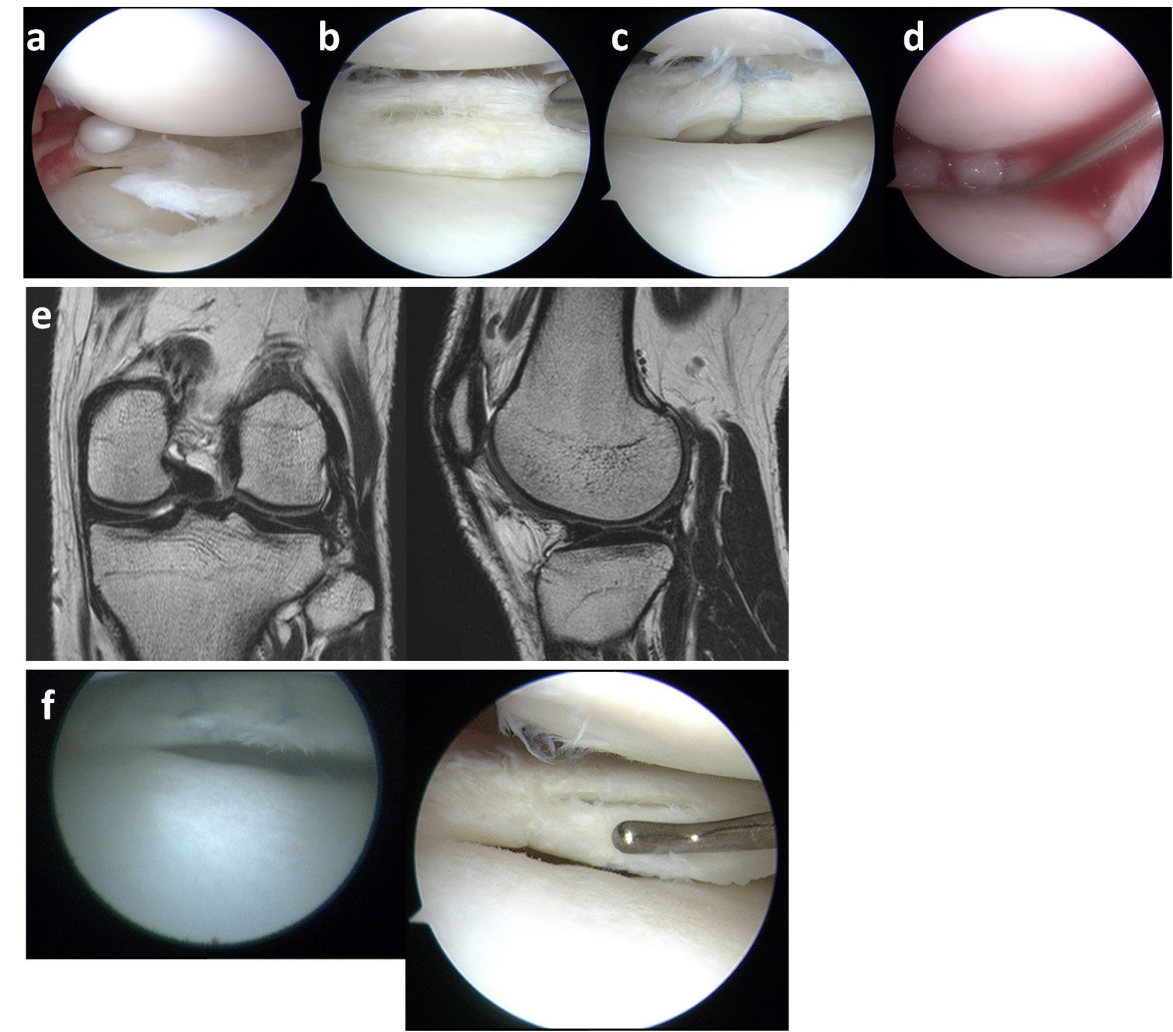
Treatment course of patient #4. (**a**) The left lateral meniscus shows a discoid meniscus with a flap tear in the posterior segment. (**b**) Following partial meniscectomy, a horizontal tear with degenerative changes is seen in the posterior segment. (**c**) Vertical sutures were placed arthroscopically using an all-inside device, followed by (**d**) P47K-WAS-MR gel application with a needle. (**e**) Postoperative T2-weighted MRI shows grade 2 healing in coronal and sagittal views. (**f**) Second-look arthroscopy shows an incompletely healed meniscus with views from the Needle Arthroscope^®^ (left) and conventional arthroscopy (right); while sutures are visible, the Needle Arthroscope^®^’s lower resolution limits probing of the repair site.

Patient #4, a national-level wrestler, had relatively positive clinical outcomes. Initially, he experienced a catching sensation in his left knee during training. Thirteen months post-injury, he underwent arthroscopic meniscal repair with SE application. During surgery, a discoid lateral meniscus with a flap tear was found in the mid-to-posterior segment (Fig. 4a). A partial meniscectomy of the flap tear was performed, followed by two vertical sutures applied to a horizontal tear in the posterior segment using an all-inside technique, along with SE application (Fig. 4b-d). The Lysholm score, KOOS subscales, and VAS score all showed improvement (Table 3). Postoperative T2-weighted MRI showed a grade 2 classification (Fig. 4e), and the repair site was assessed as “incompletely healed” by second-look arthroscopy (Fig. 4f).

**Fig. 5:**
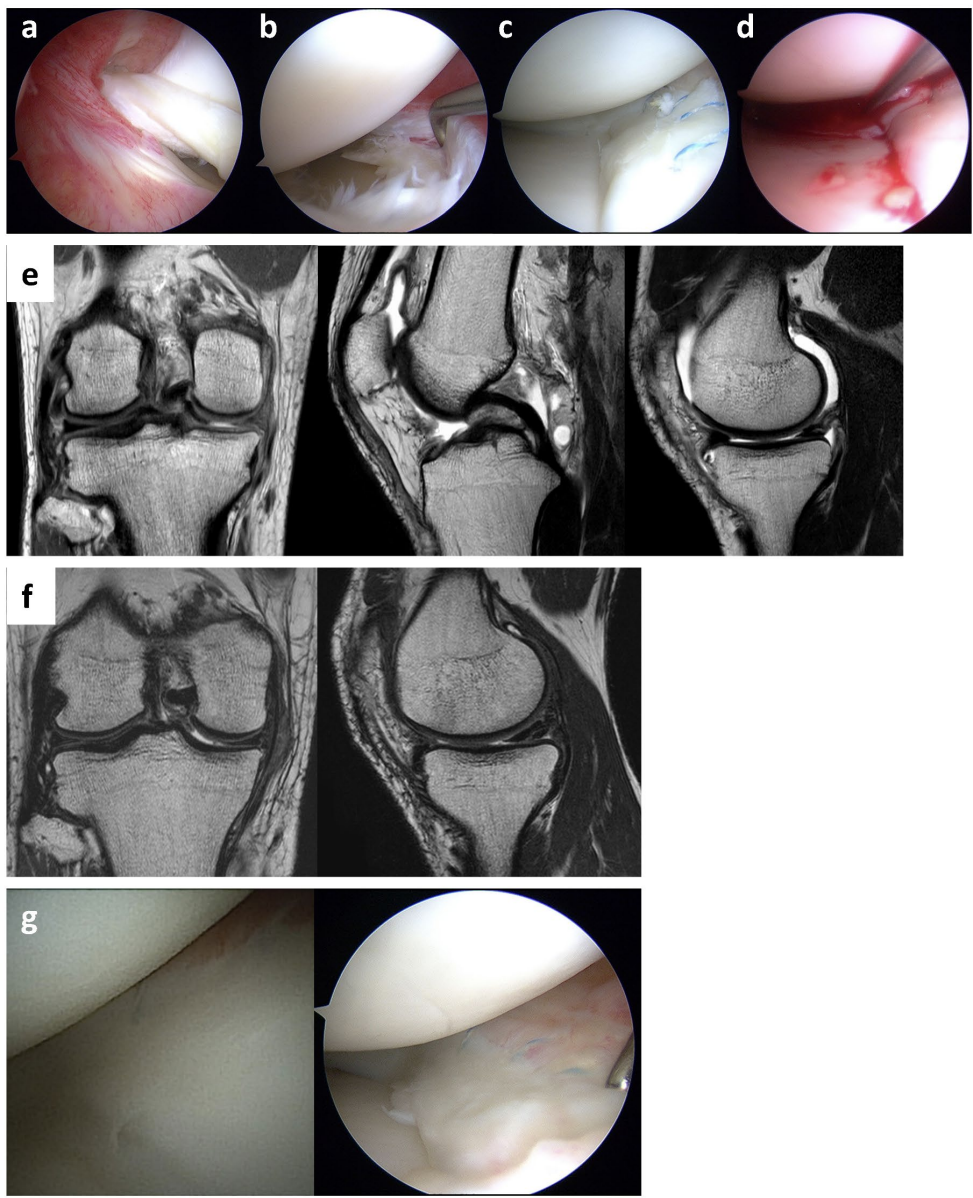
Treatment course of patient #8. (**a**) The right medial meniscus with bucket-handle tear displacement into the intercondylar region. (**b**) Arthroscopic confirmation of the tear in the avascular zone. (**c**) Inside-out sutures used for repair, followed by (**d**) P47K-WAS-MR gel application. (**e**) Preoperative T2-weighted MRI shows medial meniscus displacement in the coronal view and a double PCL sign in the sagittal view. (**f**) MRI at 3 months post-surgery indicates reduced meniscus position, graded as 2 on T2-weighted imaging. (**g**) Second-look arthroscopy reveals complete healing with views from the Nanoscope^®^ (left) and conventional arthroscope (right).

Patient #8 sustained a knee injury during judo practice. Five months later, arthroscopy revealed a bucket-handle tear in the medial meniscus (avascular zone) (Fig. 5a and b). The repair involved two all-inside sutures and 10 inside-out sutures, followed by SE application (Fig. 5c and d). Preoperative MRI displayed a displaced medial meniscus in the intercondylar region on the coronal image, with a double PCL sign on the sagittal T2-weighted MRI (Fig. 5e). Postoperative T2-weighted MRI showed a reduced medial meniscus position in both coronal and sagittal images, classified as grade 2 (Fig. 5f). The Lysholm score, KOOS subscales, and VAS score showed improvement (Table 3). Second-look arthroscopy confirmed complete healing 3 months post-surgery (Fig. 5g).

## Discussion

This single-center, open-label, uncontrolled, single-arm, first-in-human study aimed to evaluate the safety and efficacy of SE (P47K-WAS-MR gel) in patients undergoing meniscus repair for meniscal injuries. Our findings confirm the safety of the P47K-WAS-MR gel, its intra-articular application method, and the dosage (2 mL). At 3 months postoperatively, arthroscopic evaluation demonstrated healing at the repaired site, supporting SE’s potential therapeutic effect.

The primary endpoint, safety, was confirmed by the absence of serious adverse events directly related to SE. Although all eight patients experienced adverse events, these were largely surgery-related and not attributed to SE. All events resolved by the final observation and clinical assessments – including physiological and laboratory test results – showed no concerning changes, reinforcing SE’s favorable safety profile. The application technique, using gel-form SE and a needle for intra-articular delivery, proved safe and feasible without complications. The chosen 2 mL volume presented no additional risks, as no serious adverse events occurred.

A notable feature of this trial was the evaluation of all patients using second-look arthroscopy at 3 months post-surgery. In this study, a relatively less invasive needle arthroscope^25–27^ was used to evaluate the repair site. This approach is valuable because it provides an accurate, direct assessment of the postoperative course with minimal invasiveness. Previous studies have shown that needle arthroscopy has superior diagnostic ability for meniscal injuries compared with MRI^25,26,28–30^. Our results revealed that healing assessments by MRI did not always match direct arthroscopic observations. While the needle arthroscope initially aimed to reduce invasiveness by avoiding skin incision, its limited image quality and inability to palpate the repair site were found inadequate for observation. Therefore, a conventional arthroscope was used after needle scope observation. No adverse events were associated with arthroscopy, and conventional arthroscopy as an endpoint at 3 months post-surgery is recommended for future exploratory studies. Additionally, this endpoint is beneficial for patient care, as activity can be adjusted based on the repair status 12 weeks postoperatively. As SE is known to be absorbed in vivo within approximately 8 weeks^24^, its effect on meniscus repair at 3 months postoperatively is considered negligible.

Regarding clinical outcomes, all patients showed significant improvement in the Lysholm score (*P* < 0.05). Despite the limited sample size, there was no substantial difference in VAS scores between patients with incomplete and complete arthroscopic healing; overall, VAS scores significantly improved (*P* < 0.05). Factors associated with osteoarthritis – such as meniscal degeneration, tear morphology, and varus limb alignment – were among the exclusion criteria. In one of eight patients (aged 47 years) with a horizontal tear, pain improvement was unclear, potentially due to underlying degenerative changes. Continued follow-up is necessary to confirm SE’s therapeutic efficacy for degenerative conditions.

Arthroscopic assessment at 3 months was considered a conservative predictor of long-term success, establishing that the minimum requirement for SE’s performance is complete arthroscopic healing at 3 months. However, animal studies have not yet clarified how failures, such as those from mechanical stress during rehabilitation, impact healing outcomes, and symptoms may develop over time due to repair failure, leading to potential reoperation. Alhamdi et al. reported a high failure rate (65%) in medial meniscus bucket-handle tears with a mean follow-up of 6.4 years^31^. They observed that partial resection was smaller at reoperation than at the initial procedure and recommended suture repair as the first option, even for tear morphologies with high failure rates. Consequently, achieving healing with SE application at 3 months could minimize damage even if a tear recurs during long-term follow-up.

In this study, SE was applied solely to the white-white zone of meniscal tears, which is challenging to manage due to its avascular nature^32^. However, healing was confirmed arthroscopically at 3 months. Bucket-handle tears generally have a high failure rate, with a recent systematic review reporting approximately 15%^11^. In our study, two bucket-handle tears were among the eight cases, and both achieved complete healing on second-look arthroscopy. Further long-term follow-up is needed to determine the durability of the repaired site after SE application in the meniscus’s avascular region. Specifically, the findings of this study are important for understanding factors, such as changes in mechanical loading, that may influence long-term healing and re-tear risks. Future follow-up data will provide insights into key factors affecting re-tear risk.

This study has some limitations. First, it lacks a histological evaluation of the repaired tissue. In a rabbit model of meniscus injury, SE-treated areas were filled with cartilage-like tissue, and tissue repair was confirmed at 12 weeks postoperatively. Second, the observation period was relatively short (3 months), as the study primarily focused on safety and verifying the absence of serious adverse events after SE application. Although healing was observed, long-term outcomes remain unknown and are currently under follow-up.

In conclusion, this trial confirmed that SE, when applied for the first time in a human joint, did not cause severe adverse events and promoted the healing of meniscal injuries. SE gel is minimally invasive, can be easily applied to meniscus sutures using arthroscopy, and is compatible with any suture technique. As an off-the-shelf product, it is convenient for surgeons and minimally invasive for patients. Therefore, SE may be a valuable option for accelerating meniscal healing in clinical practice. Ensuring complete healing could reduce reoperations, preserve meniscal function over the long term, and potentially prevent KOA.

## Methods

### Study design

This was a single-center, prospective, single-arm clinical trial with a 12-week follow-up, culminating in an arthroscopic examination at 3 months. The study was conducted at Hiroshima University Hospital between July 20, 2022, and September 31, 2023, and the study protocol was approved by the Institutional Review Board of Hiroshima University (approval number 54002). The clinical trial notification was submitted to the Japanese Pharmaceuticals and Medical Devices Agency on June 14, 2022. The study adhered to the ethical principles of the Declaration of Helsinki and the International Conference on Harmonization Good Clinical Practice Guidelines. All participants provided written informed consent. The study was registered in the Japan Registry of Clinical Trials on 14/09/2022 (registration number: jRCT2062220056) and Patient #1 was registered on 01/10/2022.

### Inclusion criteria

Participants met the following criteria: (1) age 8 to < 60 years at the time of consent (based on SE’s intended use for discoid lateral meniscus treatment in younger patients); (2) MRI-confirmed meniscal injury involving the avascular white-white zone^32^, either alone or with anterior cruciate ligament injury; and (3) persistent pain despite conservative treatment with physical therapy or analgesics.

### Exclusion criteria

Exclusion criteria encompassed: (1) body mass index (BMI) ≥ 50 kg/m^2^; (2) history of total meniscectomy; (3) prior ligament reconstruction or osteotomy of the affected knee within 1 year; (4) intra-articular injection into the affected knee within 2 months before consent; (5) knee deformity > 10° valgus or varus from the normal femorotibial angle (FTA) (FTA 175°); (6) autoimmune arthritis (e.g., rheumatoid arthritis), sepsis, gout, pseudogout, pyogenic arthritis, or secondary arthritis; (7) diabetes mellitus; (8) known allergies to silk, anesthetics, or antiseptics; (9) positive for hepatitis B surface antigen, hepatitis C virus antibody, or human immunodeficiency virus antigen/antibody; and (10) participation in other clinical trials within 6 months before screening.

### Patient and public involvement

Patients and the public were not involved in developing the research questions; however, four volunteers reviewed the study protocol and provided informed consent.

### Recruitment procedure

**Fig. 6:**
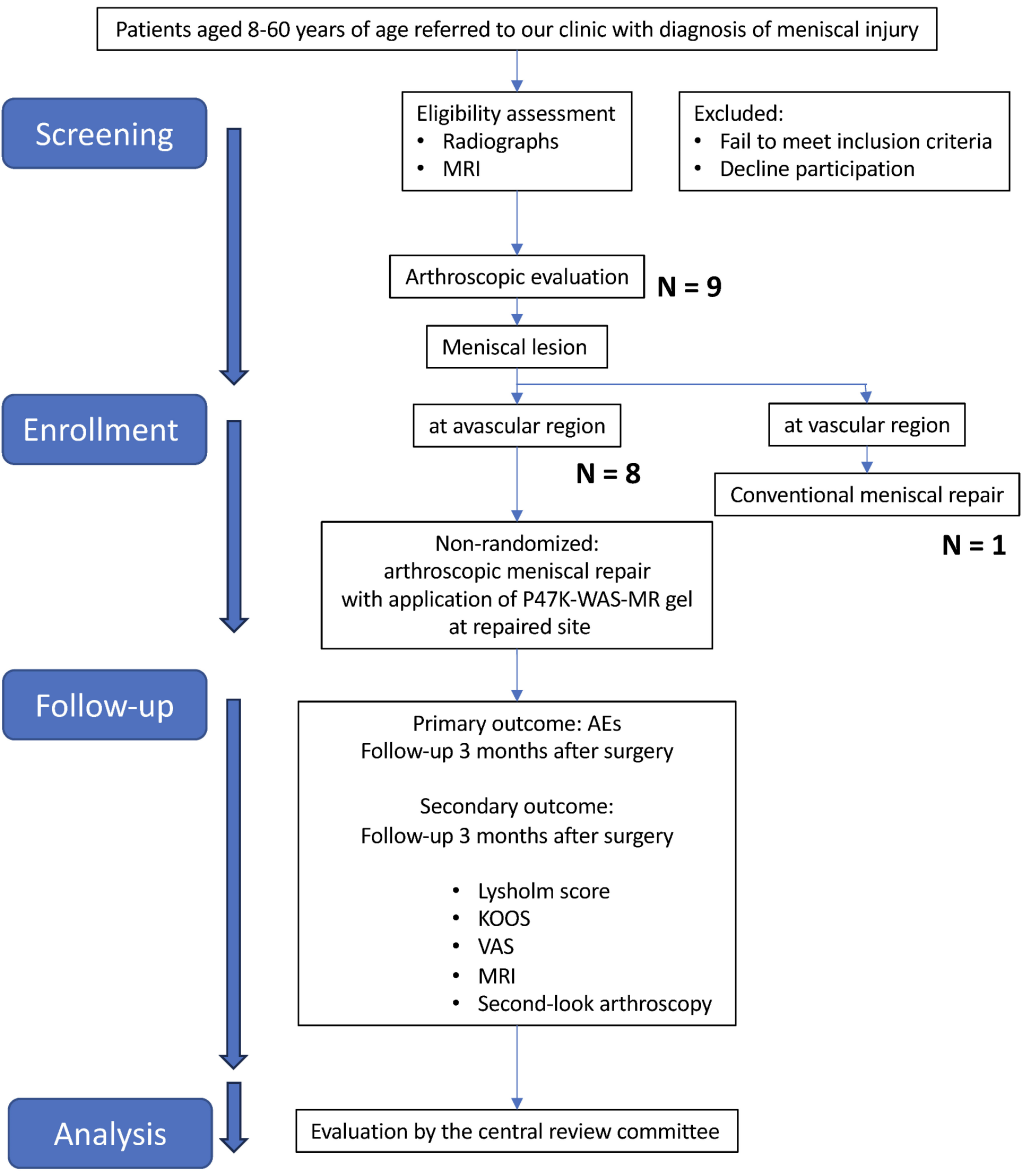
Flow chart of the study design.

Eligibility was assessed using clinical information, including patient background, clinical symptoms, physical examination findings, radiography, MRI scans, and details of concomitant medications or adjunctive therapies. Final registration occurred once all inclusion and exclusion criteria were met. The trial flow is illustrated in Fig. 6.

### Meniscal repair planning

Arthroscopic examination was performed under general anesthesia, with surgeons confirming the morphology, location, and vascularity of meniscal tears within the red-red, red-white, and white-white zones based on preoperative MRI. Meniscal repair involved suturing the tear, including the avascular area, using both inside-out and all-inside sutures. For all-inside sutures, FastFix (Smith & Nephew, Andover, MA; all-inside repair device with an anchor) and Scorpion (Arthrex, Naples, FL; all-inside suture instrument) devices were used.

### SE Preparation

**Fig. 7:**
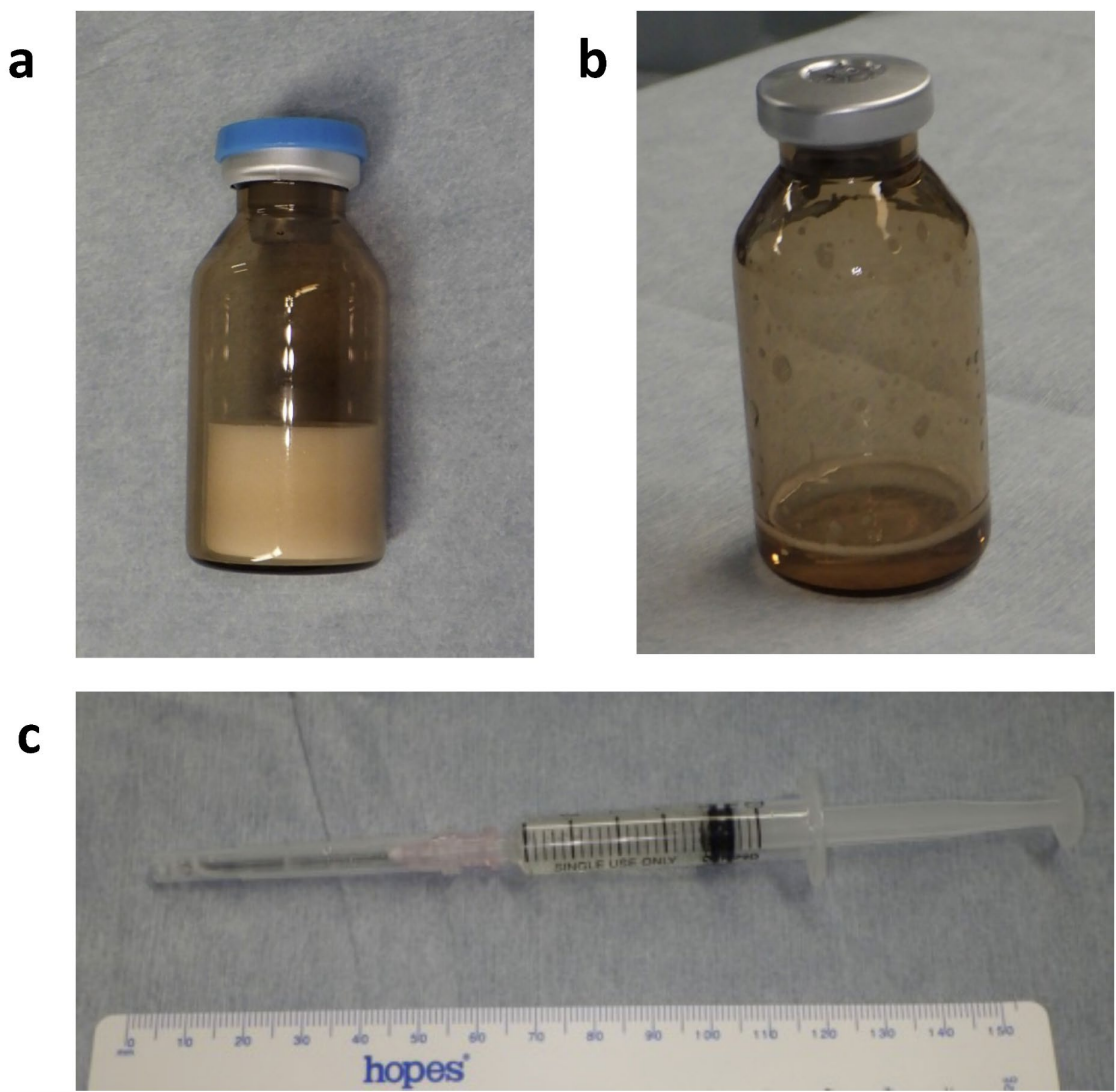
Preparation process of P47K-WAS-MR gel. (**a**) Bottled P47K-WAS-MR sponge. (**b**) Dissolution of SE sponge in 2 mL of saline. (**c**) SE gel drawn into a syringe.

The investigational device, SE (P47K-WAS-MR sponge), was manufactured by Sanyo-Kasei Kogyo, Kyoto, Japan, under good manufacturing practices conditions. The lyophilized P47K-WAS-MR sponge was stored in vials at room temperature, away from heat, humidity, and direct sunlight until use (Fig. 7a). In the operating room, 2 mL of saline was added to each vial to dissolve the sponge into a gel (Fig. 7b**)**, which was then drawn into a syringe and kept at room temperature until application (Fig. 7c).

### Arthroscopic SE application procedure

After meniscal repair, a 21 G or 23 G needle was guided arthroscopically to the repair site, and P47K-WAS-MR gel was applied, with placement visually confirmed (Fig. 1**)**.

### Postoperative follow-up

Postoperative care followed standard practices. The Lysholm score^33^ and VAS score for pain^34^ were recorded preoperatively and at 3 months post-surgery. MRI and second-look arthroscopy were conducted at 3 months to assess repair site healing. Any complications and adverse events were documented.

### Outcome measures

The primary outcomes were safety and technical feasibility of the procedure. Exploratory efficacy was assessed at 3 months with second-look arthroscopy and clinical scores.

### Primary outcome

The primary endpoint was SE safety, defined by the incidence of adverse events attributed to SE. All adverse events were recorded after SE application and categorized as “severe,” “moderate,” or “mild.” Severe events were defined per the ICH E2 criteria (e.g., death, life-threatening events, hospitalization, disability, or congenital anomalies).

### Secondary outcomes

Secondary endpoints included clinical score evaluations using the Lysholm, KOOS, and VAS scores, as well as repair success assessed through imaging modalities.

#### Clinical scores

The Lysholm scale consists of eight items that assess limping, support, locking, instability, pain, swelling, stair climbing, and squatting, with total scores ranging from 0 to 100. A score of 0 indicates the most severe symptoms, while 100 reflects the absence of symptoms. The KOOS includes five subscales: “symptoms,” “pain,” “activity of daily living,” “function in sports and recreation activities,” and “quality of line (QOL),” each containing multiple questions to comprehensively assess knee function and patient experience. Pain severity was evaluated using the VAS, providing a standardized, numerical indication of the patient’s pain level.

#### MRI evaluation

Postoperative MRI was performed to assess meniscal repair sites using a 3.0-T scanner (Canon Medical Systems, Vantage Titan 3T, Japan). T2-weighted coronal and sagittal images were acquired, and signal changes were graded using the Crues 3-stage classification system^35^: Grade 0 indicates a normal meniscus with low signal intensity; Grade 1 represents an irregularly marginated intrameniscal signal that does not abut or communicate with the articular surface; Grade 2 describes a linear signal not abutting or communicating with the articular surface, and Grade 3 denotes a linear signal intensity that abuts or communicates with the articular surface, indicating an “unhealed” status. MRI scans were conducted at the 3-month postoperative mark. A blinded, third-party image evaluation committee member centrally assessed the healing status.

#### Second-look arthroscopy

An endpoint of exploratory efficacy was the rate of healing failure at the repair site 3 months post-surgery. The arthroscopic second-look observation, performed under general anesthesia, utilized the Arthrex NanoScope system (Arthrex Inc., Naples, FL, USA) or a Needle Arthroscope (Smith & Nephew KK, Tokyo, Japan), followed by a conventional scope. After needle arthroscopy, a conventional arthroscope was introduced through anterolateral and medial portals created based on the initial surgery. The repair site was assessed at three levels of healing: “completely healed,” “incompletely healed,” and “unhealed.” A meniscus was considered “completely healed” if the tear was covered with tissue, leaving a residual cleft of < 10% of the meniscal thickness. “Incompletely healed” indicated a residual cleft of < 50% of the meniscal thickness, while an “unhealed” meniscus was one with a residual cleft of > 50% of the thickness along the tear site^36–38^. Similar to MRI, a blinded, third-party image evaluation committee member conducted a central assessment to verify healing status using images obtained from conventional arthroscopy.

### Statistical analyses

Data analyses were performed independently by statisticians in accordance with the study protocol. Patient characteristics were expressed as mean ± standard deviation (SD) or median (range), as appropriate.

For the primary outcome, adverse events related to the P47K-WAS-MR gel application and device failures were described for each participant, including event name (classified per MedDRA/J), onset and resolution times, outcome, severity, treatment, and causal relationship to the gel. All patients who received the gel were included in the exploratory efficacy analysis. The rate of unhealed repairs at 3 months post-intervention was assessed based on MRI and arthroscopic findings.

Changes in clinical scores, including Lysholm and VAS pain scores, were analyzed by comparing preoperative values to those at the 3-month postoperative mark.

## Data Availability

The datasets generated and/or analyzed during the current study are not publicly available. Masakazu Ishikawa, as the corresponding author, should be contacted for data availability.
